# The Toxicity of Amyloid *β* Oligomers

**DOI:** 10.3390/ijms13067303

**Published:** 2012-06-13

**Authors:** Li Na Zhao, HonWai Long, Yuguang Mu, Lock Yue Chew

**Affiliations:** 1 School of Physical and Mathematical Sciences, Nanyang Technological University, 21 Nanyang Link, 637731, Singapore; E-Mails: zhao0139@e.ntu.edu.sg (L.N.Z.); leon0101@e.ntu.edu.sg (H.W.L.); 2 High Performance Computing Centre, Nanyang Technological University, 50 Nanyang Avenue, 639798, Singapore; 3 School of Biological Sciences, Nanyang Technological University, 60 Nanyang Drive, 637551, Singapore

**Keywords:** molecular dynamics simulation, Alzheimer’s disease, amyloid *β* peptide, amyloid *β* oligomer toxicity mechanism, curcumin

## Abstract

In this review, we elucidate the mechanisms of A*β* oligomer toxicity which may contribute to Alzheimer’s disease (AD). In particular, we discuss on the interaction of A*β* oligomers with the membrane through the process of adsorption and insertion. Such interaction gives rises to phase transitions in the sub-structures of the A*β* peptide from *α*-helical to *β*-sheet structure. By means of a coarse-grained model, we exhibit the tendency of *β*-sheet structures to aggregate, thus providing further insights to the process of membrane induced aggregation. We show that the aggregated oligomer causes membrane invagination, which is a precursor to the formation of pore structures and ion channels. Other pathological progressions to AD due to A*β* oligomers are also covered, such as their interaction with the membrane receptors, and their direct versus indirect effects on oxidative stress and intraneuronal accumulation. We further illustrate that the molecule curcumin is a potential A*β* toxicity inhibitor as a *β*-sheet breaker by having a high propensity to interact with certain A*β* residues without binding to them. The comprehensive understanding gained from these current researches on the various toxicity mechanisms show promises in the provision of better therapeutics and treatment strategies in the near future.

## 1. Introduction

The pathogenesis of Alzheimer’s disease (AD) is characterized by the aggregation of amyloid *β* peptides leading to extracellular senile plaque, and the formation of intracellular neurofibrillary tangle by the hyperphosphorylated tau protein. These structures have the detrimental effects of causing a significant loss of neurons and synapses, which gives rise to the state of Alzheimer’s disease. Amyloid *β* peptide (A*β*) is cleaved from the amyloid precursor protein (APP) and it usually possesses 36–43 amino acids. A*β* peptides are known to self-assemble into dimer, trimer and higher-order oligomers, which are believed to be the main source of toxicity by causing the death of neurons [1,2]. The mechanism of the toxicity has been studied extensively from both the experimental and theoretical perspectives which are summarized here: (1) activation of inflammatory effects by interacting directly with the membrane [3]; (2) induction of oxidative stress [4] through the formation of metal-A*β* complex [5,6]; (3) disruption of membrane receptors’ function by intimate binding [7]; (4) formation of membrane pore [8–11] and alteration of ionic homeostasis [12,13] across the membrane; and (5) modification of the structure of certain DNA by the process of attachment [14].

In this review, we shall focus on the oligomers-membrane interaction and discuss the underlying mechanisms of the toxicity and their consequences. In this respect, we shall first exhibit the structure of small oligomers in aqueous environment, the process of A*β* adsorption, insertion, aggregation and ion channel/pore formation, as well as a brief review on the toxicity of intra-neuronal A*β*. We then bring out the relationship between A*β* and the membrane receptors, and discuss on the contribution of A*β* to oxidative stress. The secondary structure evolution of A*β* is then highlighted by means of the *α*-helix to *β*-sheet phase transition from the point of view of statistical physics of coarse-grained models. Finally, we examine into the potential inhibitory influence of the curcumin molecules on A*β* oligomers formation from the viewpoint of molecular dynamic (MD) simulations, with the inclusion of relevant experimental evidences and validations.

## 2. A*β* Oligomers in Aqueous Environment

Presently, there is intense interest in elucidating the structures of A*β* oligomers. Unlike amyloid fibril, whose structural understanding has been developed over the past decades, less is known on the structures of A*β* oligomers in aqueous environment. A mature A*β*_1–42_ amyloid fibril is known to consist of a *β*-strand-turn-*β*-strand motif, which is adopted by its residues 18–42 with the *β*-sheet being in a parallel, in-register organization, while its residues 1–17 are mainly in the form of a disordered structure [15]. It is formed by the nucleation-dependent self-assembly of A*β* [16] via a series of cascade neuropathogenic process [17]. The *β*-sheet-rich mature amyloid fibril and the monomeric A*β* are found to be far less toxic than the soluble A*β* oligomers.

Although there is limited experimental information on the structures of A*β* oligomers due to its propensity to form soluble aggregates in comparison to the amyloid fibrils, improved understanding has been achieved through computer simulations based on REMD, MD and Monte Carlo approaches [18–24]. These computations investigate into the formation and conformational properties of small A*β* oligomers from dimers to hexamers (in particular, dimers). They serve to explore into the nature of the toxicity of these oligomers and to complement the limitation of experiments in capturing the transient oligomeric states. These studies have revealed that A*β*_1–42_ oligomers are quasi-spherical in shape with a hydrophobic core and a hydrophilic surface, with enhanced exposure to the solvent at the region D1 to D7 of its N terminal. This indicates that oligomerization is driven by hydrophobic effects since it is more favorable energetically for the hydrophobic residues to hide away from the solvent and to form inter-molecular contacts with each other, although electrostatic forces are also known to play an important role. The identical interaction governing the oligomerization and fibrillization may explain the similar cross-*β* structure that is found in the A*β* oligomers (especially the toxic A*β*_1–42_ fibrillar oligomers) and the mature amyloid fibril. However, in contrast to the amyloid fibril, antiparallel-*β* sheets are observed in the oligomers instead of the fibrill-like parallel, in-register *β*-sheet structure [24]. During dimerization, it is also observed that there is a consistent reduction in the *α*-helical content with an increase in *β*-sheet structure. The residues ILE-41 and ALA-42 and the formation of salt-bridge between D23-K28 are found to be important in the dimer formation [23]. Notably, the free energy landscape of A*β*_42_ dimers is found to be complex and broad, indicating its greater tendency to form hydrophobic contacts and *β*-sheet structures relative to the dimers of A*β*_40_ [22,23]. More importantly, it is uncovered that a small difference in the A*β* primary structures can lead to significant differences in the resulting oligomer structures. This implies that to anticipate and fathom the associated toxicity properties of the eventual oligomer is not a straightforward task.

## 3. A*β* Adsorption and Insertion Mechanism

The influence of membrane surface on the adsorption and aggregation of A*β* peptides have been investigated on solid surfaces, monolayer bilayers, self-assembled monolayers (SAMs), implicit membrane models, and models that mimic membrane structures [25–30]. The general observation is that solid surfaces promote the self-assembly of A*β* peptides. The main driving forces for the association have been attributed to dehydration effects and electrostatic interactions. A detailed review on A*β* interaction with solid surface can be found in Reference [25]. On the other hand, the adsorption process of A*β*, ranging from dimer to hexamer, towards a self-assembled monolayers surface which is capped by the COOH, CH_3_ and OH groups, is also explored [26,27]. These researches have determined that the strength and extent of the adsorption on the SAMs surface are related to the binding sites. Furthermore, the A*β* is observed to change its conformation and reorient itself in order to adopt a more energetically favorable association during the adsorption process. However, the conformational change of the oligomers is found to be slowed down by the SAMs relative to oligomers that are placed solely in water. Extensive MD simulation studies have found that trimers and tetramers have well-preserved *β*-sheet structures that act as seed for future oligomerization and fibrillization. The hydrophobic effects, electrostatic interactions and water-mediated dewetting transition have been highlighted as the main driving forces behind these aggregation process [16,31–33].

Implicit membrane model with short A*β* fragments has been used to investigate the interaction of A*β* with the membrane and the folding process of A*β* in the membrane environment [28]. An interesting observation in this study is that the *C*-terminus of A*β* (25–35) first forms an *α*-helical structure in the hydrophilic region of the membrane before the A*β* attempts to knock into the hydrophobic membrane core through its hydrophobic residues. The study of A*β* peptides adsorption on different membrane monolayers has revealed that the adsorbed A*β* exhibits *β*-sheet structure with its orientation aligned parallel to the air-water interface and the lower pressure lipid surface [29]. The association of A*β* with the monolayer has the effect of increasing the surface pressure of the layer. On the other hand, the interaction of A*β* with the membrane bilayer affects both the order and fluidity of the bilayer. This is observed when the two fragments of A*β*_1–28_ and A*β*_25–40_ are incorporated into the bilayer. While the hydrophobic group of A*β*_25–40_ is observed to locate inside the hydrophobic core region, A*β*_1–28_ shows a greater propensity to interact with the hydrophilic region of the membrane [30]. In comparison to A*β*_1–28_, A*β*_25–40_ is found to induce larger membrane perturbation and alteration.

**Figure 1:**
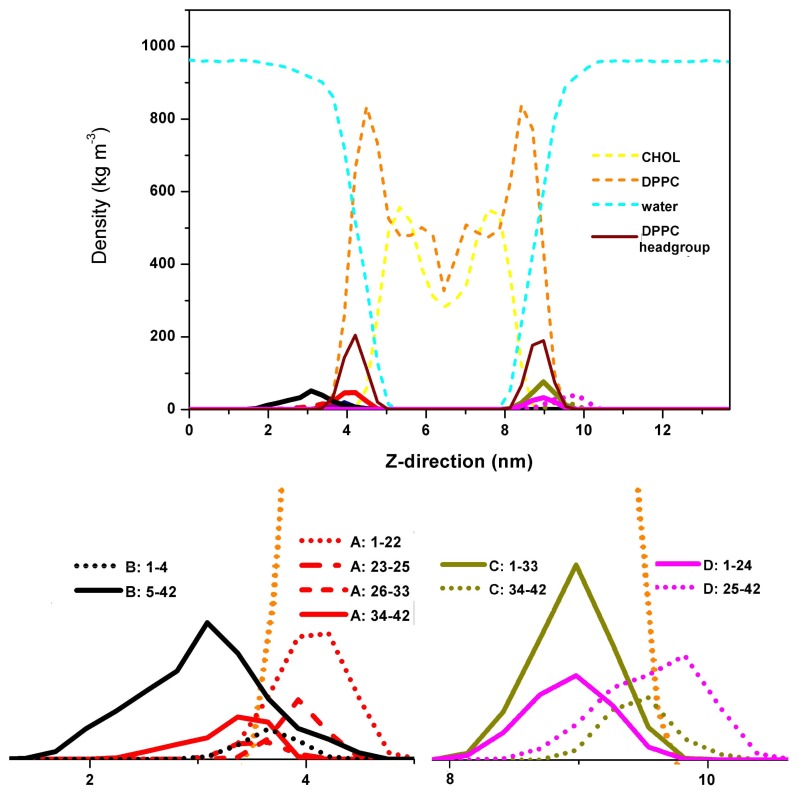
Partial density function along the *Z*-direction.

**Figure 2:**
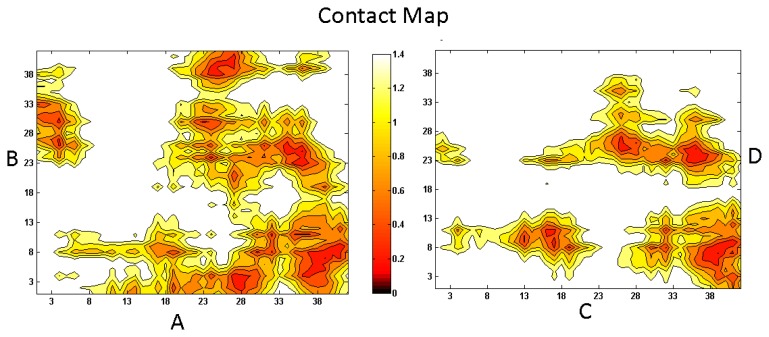
The residue contact map of each dimer. The coloring scheme is based on the inter-residue distance.

In our investigations, however, we have uncovered that while the *C*-terminus of the A*β* peptide remains outside the membrane, the *N*-terminus tends to bury inside the DPPC headgroup as a result of the strong protein-lipid interactions. We have considered two pre-formed A*β* dimers in our simulations, which consist of 4 A*β* peptide chains: A, B, C and D. They are represented by the GROMOS96 43a1 force field. These peptides are rich in *β*-sheet contents and they are placed at each side of the pre-equilibrated dipalmitoylphosphatidylcholine (DPPC) and cholesterol (CHOL) mixed bilayer model, with an initial 2 nm distance from the membrane surface. The membrane model is represented by the 43A1-S3 parameter set [34–38]. We have used 31*β* 777 water molecules with the addition of 103 Na^+^ and 95 Cl^−^ ions to neutralize the system and reach the ions concentration of 0.1 mol/L. The temperature was kept at 323 K using the Nosé–Hoover coupling scheme [39,40]. The linear constraint solver (LINCS) algorithm [41] with a 2 fs integration time step was employed to constrain all the bonds in the simulation. Our results have indicated that the dimers mainly adsorb and reside on the membrane surface during the 1.2 *μ*s simulations. The partial density functions of the dimers, water molecules, and the constituents of the mixed bilayer during the last 100 ns are given in Figure 1. The result here shows that the *N*-terminus of chain A, from residues 1–22 and 26–33, is fully inserted into the DPPC headgroup, while the *C*-terminus remains outside the membrane. On the other hand, chain B is observed to interact with chain A and has correspondingly less interactions with the membrane. Similar to chain A, the *N*-termini of chains C and D from 1–33 and 1–24 respectively are totally buried within the DPPC headgroup. Their *C*-termini, however, are found to stay outside the membrane. From Figure 2, we see that the dimer which is made up of chains A and B is more stable than the dimer formed by chains C and D. Furthermore, it is observed that A*β* peptides adsorption onto membrane surface do not always have stronger protein-protein interaction within the dimer. The perturbation of the bilayer due to the association of A*β* dimers is also found to be insignificant.

## 4. Membrane-Mediated A*β* Aggregation Mechanism

The toxicity of A*β* oligomers originates from the cleavage of APP with subsequent deposition of excessive A*β* peptides on the membrane surface, and to the cleaved peptides that fail to exit and release from the membrane into the extracellular space which form intracellular neurofibrillary tangle. Thus, a good understanding on the toxicity mechanisms requires knowledge on the possible structures of the transmembrane parts of the peptide and the detailed cleavage scenario of APP [42]. In particular, in order to find the possible aggregation behavior inside the membrane [18], it is necessary to examine the interaction of A*β* with the membrane, and cumulative evidence has suggested that elevated cholesterol level in the membrane plays an important role in increasing the risk of Alzheimer’s disease [43]. Indeed, research has found that A*β* is produced in the cholesterol-rich areas, which is also known as the lipid rafts. Such raft-like heterogeneous membrane environment which consists of ganglioside and cholesterol [44–46] has been extensively used to study the interaction of A*β* with the membrane environment. Study has proposed that lipid rafts accelerate the aggregation of A*β* [47], with the controversial results of cholesterol inhibiting the interaction of A*β* with the gangliolipids [48] while promoting the interaction of A*β* with 1-palmitoyl-2-oleoylphosphatidylcholine (POPC) bilayer[49]. Our research has revealed that the interaction between A*β* and lipids has facilitated the aggregation of the A*β* peptides. However, the interaction between A*β* and the cholesterol is inversely correlated with the extent of the peptide-peptide interactions [18]. The depletion of cholesterol or gangliosides has been shown to significantly reduce the amount of A*β* and its accumulation [46,50]. In fact, the aggregation of A*β* to fibrills is mediated by the gangliosides on the lipid rafts, where a transition from the alpha-helix-rich conformation to the *β*-sheet-rich conformation is observed. Thus, the constituents of the raft-like membrane strictly control the amyloid formation [51].

**Figure 3:**
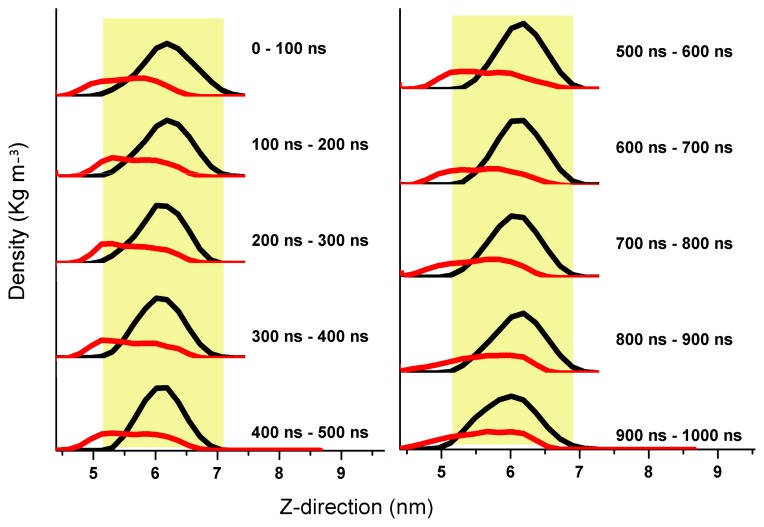
The density function of A*β* in the mixed bilayer environment along the Z-direction. Residues 1–27 are indicated by black line, and residues 28–42 by red line. The light yellow background shows the DPPC headgroup region. For detailed system information see Reference [18].

To gain a better understanding on the underlying interaction mechanism between A*β*, cholesterol and lipids, and the aggregation process of A*β* in a raft-like environment, we have arranged three full-length A*β* peptides aligning in a parallel configuration in the vicinity of a DPPC and cholesterol mixed bilayer, with residues 1–27 on the surface of the membrane and 28–42 inside the membrane. The detailed simulation information can be accessed from Ref. [18]. Our 1 *μ*s long simulation shows that while residues 1–27 predispose to interact with the aqueous-membrane interface region, residues 28–42 incline to remain inside the hydrophobic core region (see Figure 3) [18]. The oligomer is found to attach to the sunken raft-like membrane surface forming a conglomerate of defects and disordered cholesterol molecules. Cholesterol further enhances the pre-existing hydrogen-bond network between A*β* and DPPC and promotes the incorporation of A*β* into the membrane [52]. However, the interaction between cholesterol and A*β* competes with the A*β* peptide-peptide interactions such that cholesterol hardly facilitates the aggregation of A*β* once A*β* has been immersed into the membrane [18]. Nonetheless, cholesterol is observed to facilitate the formation of pore/channel in the membrane by binding directly to the A*β* during the adsorption process [53].

## 5. Intraneuronal A*β* Accumulation Mechanism

The intraneuronal accumulation of A*β* may precede the generation of A*β* plaque and neurofibrillary tangles formation [54], which may or may not [55] result in a series of pathological alterations like cognitive impairment, selective neuron loss, and axonopathy [56]. Since the intraneuronal A*β*_17–42_ secreted by *α*- and *γ*-secretases from APP has been suggested to be a normal product of neuronal metabolism, it is controversial as to whether its presence represents a sign of neuronal pathology. In fact, the manner in which A*β* comes to exist inside the neuron is still not well understood. One theory presupposes that the A*β*s get inside the neuron directly after cleavage. These A*β*s then lead to neuronal death. After that they are released into the extracellular space and form amyloid plaques subsequently [57]. Another theory suggests that A*β* is first being released outside the neuron before its re-uptake into the neuron through endocytosis or via membrane receptors. Several receptors have been reported to be able to mediate and internalize the A*β* located outside the neurons. These receptors are: alpha 7 nicotinic acetylcholine receptor [58,59], the low-density lipoprotein receptor-related protein 1 [60], and scavenger receptor for advanced glycation end products (RAGE) [61]. A detailed review on different uptake and internalization processes can be found in References [62,63].

Next, let us look at several aspects of intraneuroal A*β* toxicity. The accumulation of intraneuronal A*β* has been speculated to affect intracellular trafficking [64] which disrupts fast axonal transport [65]. Furthermore, results from APP/PS1KI mouse AD models indicate that the intracellular A*β* can cause early synaptic deficits, cholinergic neuron loss, and hippocampus atrophy [66–69]. There are also emerging evidences showing that the disruption of neuronal functions and survival is indirectly attributed to the intraneuronal A*β* [70].

## 6. Ion Channels/Pore Formation by the Incorporation of A*β* into the Cell Membrane

While A*β* oligomers exert toxicity from disruption of membrane integrity through altering its dielectric property to binding and activation of membrane receptors, they can also give rise to pores that leak Ca^2+^ ions which causes an elevation in cytosolic calcium. The pores formed by A*β* oligomers are much less efficient in transporting ions than other membrane pores, such as the nuclear pore, the ion channels, and the bacterial pores, which have all undergone the process of optimization during evolution [71]. Here we shall mainly focus on pores formed by A*β* oligomers on synthetic and cellular membrane based on experimental observations, as well as those generated from computational models.

Arispe *et al.* first proposed the possibility of A*β* forming an ion channel in 1992 [72] which serves to increase the level of intracellular Ca^2+^ ions. Later, several models have emerged which includes the helix-turn-helix [12] and *β*-sheet-twist-antiparallel-*β*-sheet [10,11] morphologies for the membrane-bound A*β* pore structures. The secondary structure of the membrane-bound A*β* is affected by many factors [44,73], such as the pH, the constituents and property of the membrane, peptide concentration, and others. By means of basin-hopping global optimization, the strand-turn-strand motif has been identified to be the most stable membrane-spanning structure for monomers, with tetrameric and hexameric *β*-sheet subunits constituting the pore structure [74]. Other proposal includes a hexamer-of-hexamer ion channel model with stable 36-stranded *β*-barrel in the membrane. This model is consistent with experimental observations and has been further used to explain the consequent channel selectivity [12]. Images from solid state NMR and atomic force microscopy (AFM) has revealed the presence of hexagonal annular channels in A*β* containing membrane. These images strongly suggest a pore-like assembly with 6 subunits [75] (in the case of rectangular assembly, 4 subunits in the membrane were observed). Zn^2+^ ions, as well as other small molecules like MRS2481 and its enatiomeric species, have been proposed to inhibit the toxicity by blocking the calcium-permeable channels formed by the A*β* oligomer [76–78].

## 7. The Relationship of A*β* with Different Receptors

We have discussed the role of adsorption, insertion, aggregation and ion channel pore formation as key determinants of A*β* toxicity via interaction of A*β* with the membrane. In this section, we shall extend our review to the area when A*β* oligomers serve as pathogenic ligands by binding to different receptors and inducing deterioration and loss of synapses through a redistribution of receptors, which further leads to alteration of neuronal plasticity accompanied with oxidative stress [79,80]. Indeed, it is well known that A*β* oligomers can bind to several receptors [81–86] and initiate numerous signaling cascades and surface expression regulations.

Recent studies have indicated that A*β* oligomers can induce impairment to the transduction of signal in neuronal insulin receptors [81], and suppress the activation of insulin receptor substrate [87]. Further studies have shown that the insulin receptor impairment and synaptic deterioration can be mitigated by insulin via down-regulation of the A*β* oligomers binding sites [79]. Omega-3 fatty acids and curcumin [87] are also reported to be able to prevent synaptic dysfunction and neuronal loss by suppressing the inactivation of insulin receptors due to the A*β* oligomers.

Several mechanisms have been proposed on the role of *N*-methyl-D-aspartate (NMDA) receptors in A*β* toxicity [88,89]. This includes the possibility that A*β* activates NMDA receptors directly [90] or indirectly by regulating the downstream NMDA receptors. Experimental observations have shown that both synthetic and naturally-secreted A*β* possesses the ability to reduce surface NMDA receptors, and reducing A*β* would restore the surface expression of NMDA receptors [82]. On the other hand, the activation of NMDA receptors affects synaptic A*β* generation [91,92] which further induces the elevation of intracellular Ca^2+^ and apoptosis [86]. In order to prevent the A*β* oligomer toxicity, various NMDA receptor antagonists have been suggested [90,93,94].

It is recently proposed that the natively folded cellular prion protein (PrP*^C^*), which is involved in the development of nervous system through mediation of synaptic and neuroprotective roles [95–100] and promoting neurite outgrowth [101], is an A*β* oligomer receptor and is related to AD by mediating synaptic dysfunction induced via the A*β* oligomers [102]. However, it is currently controversial as to whether the presence of PrP*^C^* is necessary for A*β* to induce synaptic dysfunction because there are experimental evidences which indicate that PrP*^C^* is not essential and has no effects on the impairment of synaptic plasticity induced by the A*β* oligomers [103,104].

A*β* has been reported to show a very high binding affinity towards the alpha 7 nicotinic acetylcholine receptors (nAChRs) [105], and it directly modulates [106] and blocks the response of these nAChRs [107]. Meanwhile, nAChRs is known to promote intraneuronal A*β* aggregation [58] and exacerbates cognitive deficits and synaptic pathology [108]. However, this view is in conflict with another report which states that the absence of nAChRs can enhance A*β* accumulation [109] and hence worsen the cognitive deficit. Nonetheless, the neuroprotective role of nAChRs by counterbalancing the toxicity of A*β* oligomers has been proposed based on experimental observation [109]. For example, drugs like 2-(2-(4-bromophenyl)-2-oxoethyl)-1-methyl pyridinium (S 24, 795), which have been assessed to be able to reduce the interaction between A*β* and nAChRs, have been shown to enhance long-term potentiation [110–112]. Interestingly, one research group has found that A*β* does not bind with nAChRs and has no direct relationship with the nAChRs expression and activity. Instead, the A*β* may affect the nAChRs indirectly by attaching to the membrane and altering the property of the membrane, which then influences the membrane receptors inadvertently [113]. Finally, note that there are other receptors like P75 neurotrophin receptor (P75NTR) [85,114,115], serpin-enzyme complex receptor (SEC-R) [116,117], receptor for advanced glycosylation end-products (RAGE) [118,119], and scavenger receptor CD36 [120–122], which bind with the A*β* oligomers.

## 8. Oxidative Stress

Patients with AD are found to show an elevated level of oxidative stress, which is mainly characterized by protein, DNA and RNA oxidation, and lipid peroxidation [123,124]. Oxidative stress may induce the overproduction of A*β* peptides through the activation of *β*-secretase [125]. The excessive A*β* peptides may aggregate into toxic oligomers which in turn initiate the free radical process. This results in new oxidative stress, accompanied with increased macroautophagy and lysosomal ensuing apoptosis [126], and any additional overexpression of oxide synthase can bring about extra neuronal damage [124]. Oxidative stress can also change the protein structure and affect its function, leading to physiological alteration and pathological induction [124,127,128]. In fact, a series of oxidatively modified proteins have been identified in the brain of AD patients [124,129–131]. Met-35 of A*β* is believed to be the key residue involved in oxidative stress and the mutation of Met-35 has been shown to reduce the effect of toxicity in A*β* [123,132,133]. There are many food and compounds, such as the walnut and turmeric extract, which are anti-oxidant in nature. These products have been reported to be able to prevent the oxidative stress induced by A*β* and its associated apoptosis [134].

**Figure 4:**
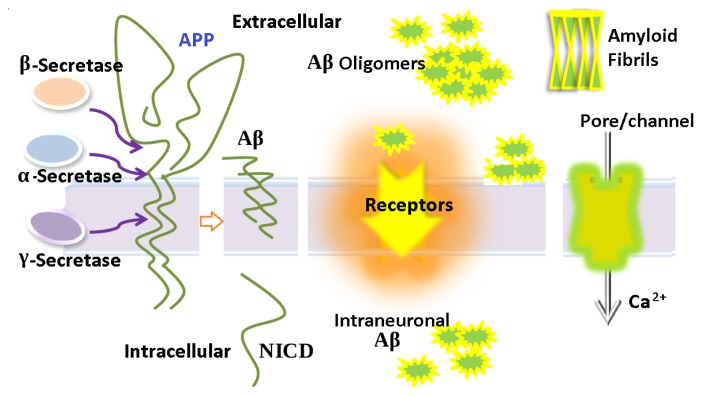
The cleavage of APP by *α*-, *β*- and *γ*-secretase and the production of A*β* peptides are shown on the left side of the figure. The following toxic mechanisms are illustrated in the figure: formation of A*β* oligomers and its further conversion to fibrils; disruption of membrane receptors; adsorption on membrane surface which alters the property of the membrane; formation of pore which causes the leakage of Ca^2+^; and the accumulation of intraneuronal A*β*.

In summary, we have discussed the various mechanisms which contribute to A*β* oligomer toxicity: adsorption, insertion, aggregation and pore formation in the membrane, as well as the interaction of A*β* with the membrane receptors and oxidative stress. The pleiotropic effects of A*β* peptides can be seen in Figure 4. Currently, there are lots of therapeutic strategies being proposed to suppress the A*β* induced toxicity, such as the *β*- and *γ*-secretase inhibitors whose function is to reduce the production of A*β* peptides. There are also other strategies to overcome A*β* oligomer toxicity, such as the aggregation inhibitors, the pore/channel blockers *etc*.; a detailed review on these therapeutic approaches is available in Ref. [135].

## 9. Secondary Structure Phase Transition

One of the important underlying mechanisms that leads to the formation of A*β* oligomer is the occurrence of secondary structure phase transition within the A*β* peptide. In particular, the transition typically involves a phase transition from an *α*-helix or a random coil to a *β*-sheet configuration [136–138]. Such structural changes or misfolding can result in a loss of normal biological functions and is well known to be a source of diseases such as the Creutzfeldt–Jakob disease, in addition to the Alzheimer’s disease [139,140]. The phenomenon of protein misfolding has led to intensive studies on the subject of protein secondary structure phase transition. A fruitful approach in these studies involved coarse-grained models which include those developed by Zimm–Bragg, Lifson–Roig, Yakubovich *et al*., Ding *et al*., Hong–Lei, Yasar–Demir, Gibbs–DiMarzio *etc*. [141–149].

In our investigation, we have explored a coarse-grained protein model based on a polypeptide consisting of backbone atoms and hard-sphere side chains. The hard-sphere side chain serves to simulate the amino acid residue. In our approach, we have defined the base unit as crank instead of residue [150,151]. Each crank has a pair of dihedral angles *φ* and *ψ*, which are the only degrees of freedom in the polypeptide. In addition, bond lengths and bond angles are being held rigid in our model. We have studied phase transition from *α*-helix to *β*-sheet, and from *β*-sheet to random coil, using this model both numerically and theoretically. While our numerical study employs the model of Conditioned Self-Avoiding Walk (CSAW), our theoretical approach is based on the Hamiltonian formulation. Note that in both of these cases, we have assumed that the non-covalent forces arise solely from the hydrogen bonds. In our theoretical analysis, we have restricted the pair of dihedral angles of a crank to only five distinct states, such that the combination of crank states can adopt only the *α*-helix, *β*-sheet and random coil conformations. Such a protein model has shown to be reliable based on our computation of the normal modes of the *α*-helix and *β*-sheet [152].

**Figure 5:**
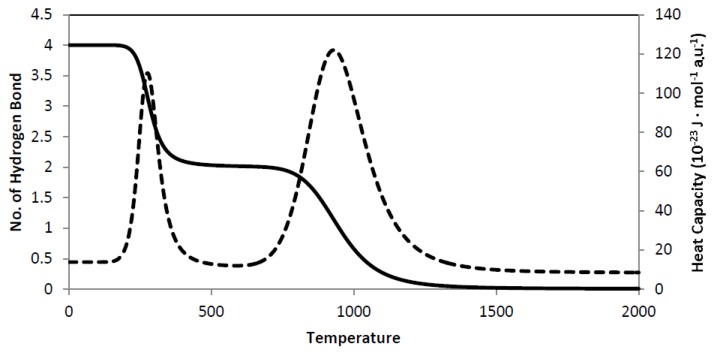
Plots of *α*-*β*-coil secondary structure phase transition for a seven crank polyalanine. Solid line represents the number of hydrogen bonds; dotted line represents the corresponding heat capacity. The transition temperatures are *T_α_*_–_*_β_* = 300 a.u, and *T_β_*_–_*_coil_* = 950 a.u., respectively.

By taking polyalanine as a prototypical example, we have plotted an *α*-*β* -coil phase transition from the ensemble average of the number of hydrogen bonds against temperature (see Figure 5 and note that 1 a.u. is to be associated with a temperature of 1 K). Such a plot allows us to predict the transition temperature of the phase transition. We have also plotted an ensemble average of the heat capacity against temperature, where we observed two significant peaks for the *α*-*β* and the *β*-coil transition. These results have demonstrated that the transitions are associated with a first-order phase transition. In our model, we have assumed polyalanine to be in the gas phase, which implies that lower transition temperatures are to be expected in an aqueous environment [153].

A key finding in both our theoretical and numerical studies relates to a biased in terms of stability for the hydrogen bond formed within the *β*-sheet and the *α*-helix. Our results show that hydrogen bonds are more stable in *β*-sheet due to a stronger hydrogen bond-to-hydrogen bond co-operative interaction [153]. This has important implication as we believe it is such a strong hydrogen bond-to-hydrogen bond interaction that leads to the aggregation behavior of the A*β* oligomer.

## 10. Curcumin

There are various small molecules, such as resveratrol [154], cyclodextrin [155,156], mitoxantrone and pixantrone [157], derivatives of Congo Red [158], curcumin and other compounds, which have been used to investigate the inhibitory effects on A*β* aggregation [159]. In fact, these molecules can serve as drugs or potential labelling agents for the diagnosis and monitoring of AD [160]. In particular, the molecule curcumin and its derivatives, with their antioxidant, anti-inflammatory, and anticancer properties, have made them very promising candidates for the treatment of Alzheimer’s disease.

**Figure 6:**
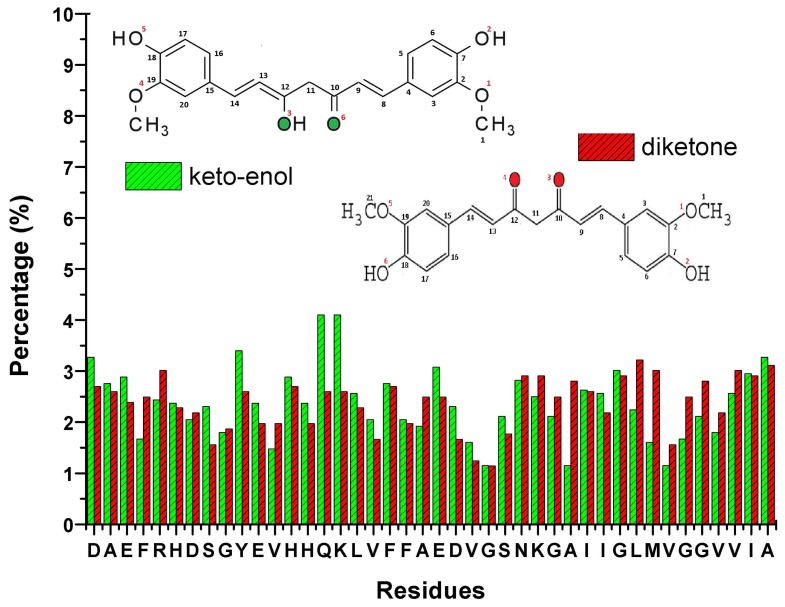
The binding propensity of curcumin towards the A*β* dimer.

**Figure 7:**
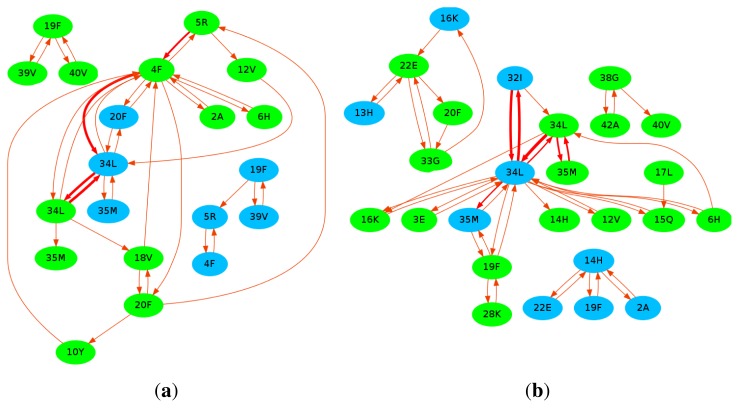
The common pathways of curcumin in the diketone (**a**) and the keto-enol form (**b**) of A*β* dimer. The blue colored domains represent the residues from chain A, while the green colored domains represent the residues from chain B. The thickness of the arrow indicates the popularity of the pathway. This figure was generated by Graphviz [165].

Curcumin can suppress the Tau phosphorylation and insulin receptor inactivation induced by A*β*. It can also improve insulin signaling and overcome cognitive deficits [87]. It is able to disrupt the existing A*β* plaques *in vivo* and partially restore the dystrophic dendrites at the same time [161]. Meanwhile, research has proposed that curcumin can reduce neurotoxicity by promoting the conversion of oligomers into fibril [162], with the inhibition of oxidative damage and tau hyperphosphorylation [163]. Solid state NMR has been employed to uncover the binding sites of curcumin on the full-length A*β* fibrils [164]. It was found that residues 12, 17–21, and the *β*-sheet structure of A*β* interact with the methoxy and/or hydroxy groups of curcumin. In order to investigate the potential binding sites of curcumin on A*β*, we had also performed 20 all-atom MD simulations for a system consisting of pre-formed A*β* dimer and curcumin molecules of both the diketone and keto-enol form. The detailed simulation information can be obtained from Reference [19]. After a total simulation time of 10 *μ*s, we plotted the binding propensity of both the diketone and keto-enol form of curcumin in Figure 6. We observed that no specific residues strongly bind to curcumin although some residues have a higher chance of forming hydrogen bonds with curcumin. Furthermore, we observed that the –OH group of curcumin has more than twice the chance of forming a hydrogen bond than the methoxy group (diketone: –OH ~ 62%, –O–CH_3_ ~ 17%; keto-enol: –OH~ 50%, –O–CH_3_ ~ 20%). Interestingly, we discover that curcumin travels along certain common pathways as it moves around A*β*. These pathways are defined to consist of at least 4 steps and are common among at least two trajectories, where each step is being made from one residue of A*β* to another. The common pathways are displayed in Figure 7. Our results show that the most popular pathways are: 34L*_B_* → 34L*_A_* → 32I*_A_* → 34L*_A_* and 34L*_B_* → 34L*_A_* → 34L*_B_* → 34L*_A_* (see Figure 7). As curcumin traverses about A*β*, we observe that it serves the role of a *β*-sheet breaker. The frequent *π*–*π* stacking interactions between its aromatic ring and the aromatic side chain of HIS, TYR and PHE are important. Although these interactions are transient, they contribute indirectly to a reduction in the *β*-sheet content in the A*β* dimer [19].

## 11. Conclusions

At present, there are still many unanswered and challenging questions for the experimentalists and theorists regarding the detailed mechanism of A*β* oligomers toxicity. Nevertheless, there are many excellent works that give a deeper insight into this area which unfortunately we are unable to cover and cite in this short review. To summarize, we list the current outstanding problems of A*β* oligomers toxicity as follow: (1) deciphering the unknown transmembrane structure of APP; (2) uncovering the configurational and structural diversity of A*β* oligomers; (3) elucidating the pathogenesis of intraneuronal A*β* accumulation; and (4) curing Alzheimer’s disease by directing the best antibodies at A*β* peptides. While there are pan-A*β* antibodies available at this point in time, these antibodies have the problem of discriminating the A*β* peptides from the much more abundant normal full-length APP, as well as the A*β* that is embedded in the APP fragments cleaved by the *β*-secretase. In conclusion, our review has demonstrated that the toxicity of A*β* oligomers arises from many factors, and in order for potential therapeutics and treatment strategy to be effective against them, extensive future research which aim to gain a more comprehensive account of the various toxicity mechanisms discussed here is both necessary and important.
